# The Prognostic Value of LncRNA SLNCR1 in Cancers: A Meta-Analysis

**DOI:** 10.1155/2021/3161714

**Published:** 2021-10-25

**Authors:** Lele Cong, Hongyan Sun, Miao Hao, Qian Sun, Yang Zheng, Xianling Cong, Rihua Jiang

**Affiliations:** ^1^Department of Dermatology, China-Japan Union Hospital of Jilin University, Changchun, China; ^2^Tissue Bank, China-Japan Union Hospital of Jilin University, Changchun, China; ^3^Science Research Center, China-Japan Union Hospital of Jilin University, Changchun, China

## Abstract

**Objective:**

This meta-analysis was performed to identify the prognostic value of SLNCR1 in multiple cancer types.

**Methods:**

Electronic databases, including PubMed, EMBASE, and Web of Science, Cochrane Library, Medline, BioMed Central, Springer, Science Direct, and China National Knowledge Internet (CNKI), were searched for relevant studies up to August 2021, and the hazard ratios (HR) and 95% confidence intervals (95% CI) were calculated to assess the relationship between SLNCR1 expression and overall survival (OS).

**Results:**

12 studies with a total of 1155 patients with 9 different types of cancers were included in this meta-analysis. The pooled HR indicates that high SLNCR1 expression represented poorer prognosis of cancer (HR = 2.11, 95% CI: 1.59–2.80, *I*^2^ = 0%, *P* < 0.00001). Additionally, high SLNCR1 expression was correlated with TNM stage (odds ratio (OR): 1.72, 95% CI: 1.08–2.74, *I*^2^ = 62%, *P*=0.02), lymph node metastasis (LNM) (OR:2.42, 95% CI: 1.61–3.64, *I*^2^ = 55%, *P* < 0.0001), and distant metastases (DM) (OR: 2.30, 95% CI: 1.50–3.55, *I*^2^ = 27%, *P*=0.0002). However, no evidence was found for a relationship between SLNCR1 expression and clinical features such as tumor size (OR: 1.71, 95% CI: 0.93–3.14, *I*^2^ = 71%, *P*=0.09), age (OR: 0.86, 95% CI: 0.68–1.08, *I*^2^ = 0%, *P*=0.19), or gender (OR: 1.07, 95% CI: 0.64–1.81, *I*^2^ = 55%, *P*=0.79).

**Conclusion:**

Our findings found that high SLNCR1 expression was associated with poor OS, advanced tumor stage, tumor size, LNM, and DM in multiple cancers, indicating that SLNCR1 may serve as a potential prognostic biomarker for cancer patients in China.

## 1. Introduction

Cancer is expected to rank as the leading cause of death and the most significant barrier to extend human life expectancy worldwide. The incidence and mortality of cancer are expected to grow rapidly with increases in population and age [1]. Although significant achievements have been made in cancer diagnosis and treatment, the five-year survival rate remains dismally low. Numerous scientists remain dedicated to find effective biomarkers for cancer patients [2].

Long noncoding RNA (LncRNA), a novel class of noncoding RNA, commonly refers to RNA transcripts greater than 200 nucleotides in length [3]. Moreover, accumulating studies indicate that LncRNAs may participate in a wide range of biological functions and act as oncogenes or tumor suppressors in cancer evolution [4, 5].

Steroid receptor RNA activator (SRA)-like noncoding RNA 1 (SLNCR1) is located on human chromosome 17q24.3. Recently, emerging evidence has revealed that SLNCR1 is aberrantly expressed in various cancers, including malignant melanoma [6], nonsmall-cell lung cancer [7], breast cancer [8], pancreatic cancer [9], and cervical cancer [10], and promotes cancer cell migration, proliferation, and invasion. However, the correlation between SLNCR1 expression and cancer prognosis remains unknown. Therefore, this meta-analysis was performed to bridge this gap in knowledge between the expression of SLNCR1 and prognosis in different kinds of cancers.

## 2. Materials and Methods

### 2.1. Literature Search Strategy and Selection

**Figure 1:**
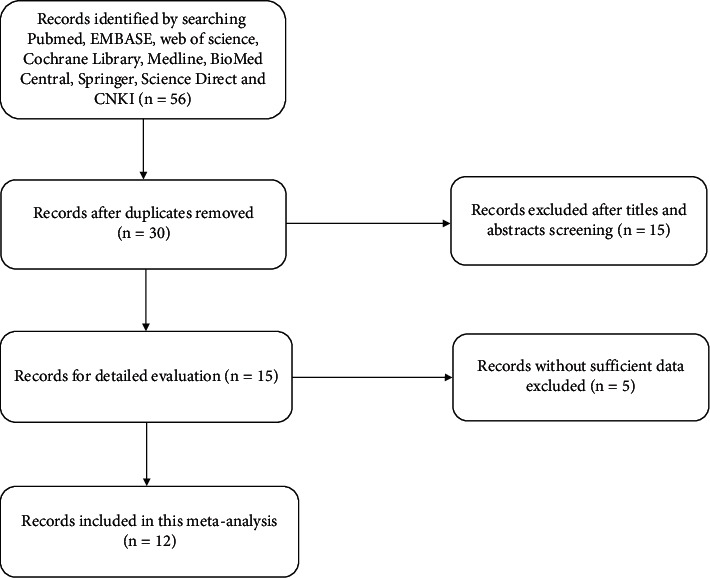
Literature screening and study selection process flow diagram.

Two of the authors conducted a systematic search of published studies to identify relevant articles on the association of SLNCR1 expression with the prognosis of cancer. English language databases, including PubMed, EMBASE, Web of Science, Cochrane Library, Medline, BioMed Central, Springer, Science Direct, and China National Knowledge Internet (CNKI), were searched for eligible studies published from inception to August 2021. The search keywords were as follows: “LINC00673” or “Lnc00673” or “lncRNA 00673,” “SLNCR1” or “SLNCR,” “ERRLR01,” “cancers,” “prognosis,” “survival” “clinicopathologic feature,” and “OS (overall survival).” In addition, the references of the relevant studies were screened to avoid omitting any potentially eligible studies. The literature screening and study selection process is shown in Figure 1.

### 2.2. Inclusion and Exclusion Criteria

The criteria for study inclusion were as follows: (1) case-control studies or cohort studies; (2) cancer was definite diagnosis by pathological examination; (3) studies examining prognostic characteristics of SLNCR1 expression in tumors, and patients were grouped in accordance with high or low SLNCR1 expression levels; (4) studies with sufficient data, including survival outcome, Kaplan–Meier curve, metastasis, and clinical features for statistical analysis.

The criteria for exclusion were as follows: (1) nonhuman studies, letters, case reports, and review articles; (2) studies without prognostic outcomes.

### 2.3. Data Extraction and Quality Assessment

Two of the authors screened all the eligible studies and completed data extraction independently. The data included the first author's name, publication year, country of origin, cancer type, sample size, age, gender, tumor size, lymph node metastasis (LNM), distant metastasis (DM), TNM stage, cutoff value, and method of detecting SLNCR1. For studies that provided only the Kaplan–Meier curve, Engauge Digitizer version 4.1 was used to extract hazard ratios (HRs) and 95% confidence intervals (95% CIs), and the method described by Tierney et al. was used to obtain survival data [11]. The quality of the included studies was evaluated by the Newcastle–Ottawa Scale [12].

### 2.4. Statistical Analysis

The strength of the association between SLNCR1 expression and the prognosis of cancer was estimated by calculating HR or odds ratio (OR) and 95% CIs. HR and 95% CIs were extracted from the Kaplan–Meier curves from published studies, and log HR and standard error were used to summarize overall survival (OS). TNM I and II were combined to indicate low tumor stage, and III and IV were combined for representing the advanced tumor stage. The OR was used to estimate the outcome. Tests for heterogeneity assumptions were checked by the Cochran *Q* statistic and *I*^2^ tests [13]. *I*^2^ < 50% and *P* > 0.05 indicated no significant heterogeneity across the studies; therefore, a fixed-effect model was used. *I*^2^ > 50% and *P* < 0.05 denoted strong heterogeneity for which a random-effect model was used for analysis. Funnel plots were utilized to assess potential publication bias. Sensitivity analyses were performed to identify individual study effects that contributed to pooled results and test the results' reliability.

## 3. Results

### 3.1. Characteristics of Included Studies

This meta-analysis encompassed 12 studies that met the inclusionary and exclusionary criteria, involving 1155 patients with eight different types of cancers: colorectal cancer [14], nonsmall-cell lung cancer [15, 16], epithelial ovarian cancer [17], gastric cancer [18, 19], thyroid cancer [20], tongue squamous cell carcinoma [21], pancreatic cancer [22], breast cancer [23, 24], and esophageal squamous cell carcinoma [25]. 56 studies associated with the prognosis and metastasis of SLNCR1 and cancers were retrieved from PubMed, EMBASE, Web of Science, Cochrane Library, Medline, BioMed Central, Springer, Science Direct, and China National Knowledge Internet (CNKI). After carefully screening the titles and abstracts, 26 studies were excluded because of duplication. Of the remaining studies, 15 were excluded because they were not case-control or cohort studies or were irrelevant to the present study, and five studies with insufficient data were excluded. Ultimately, 12 articles were selected for the present meta-analysis [14–25]. The quality assessment scores ranged from 6 to 7.

The main features of the ten studies are given in Table 1. All of the studies were conducted in China and published between 2016 and 2021. The sample sizes ranged from 35 to 229 patients. Based on the expression of SLNCR1 detected by quantitative real-time PCR, patients were divided into two groups, referred to as high and low SLNCR1 expression groups. 7 [14, 18–20, 22–24] studies focused on the relationship between SLNCR1 expression and OS, and 10, 5, 10, 12, 11, and 8 on relationships with LNM [14, 15, 17–24], DM [14, 18, 20–22], TNM [15, 17–25], age [14–25], tumor size [14–21, 23–25], and gender [14–16, 18–21, 25], respectively.

### 3.2. Association between SLNCR1 Expression and OS

**Figure 2:**
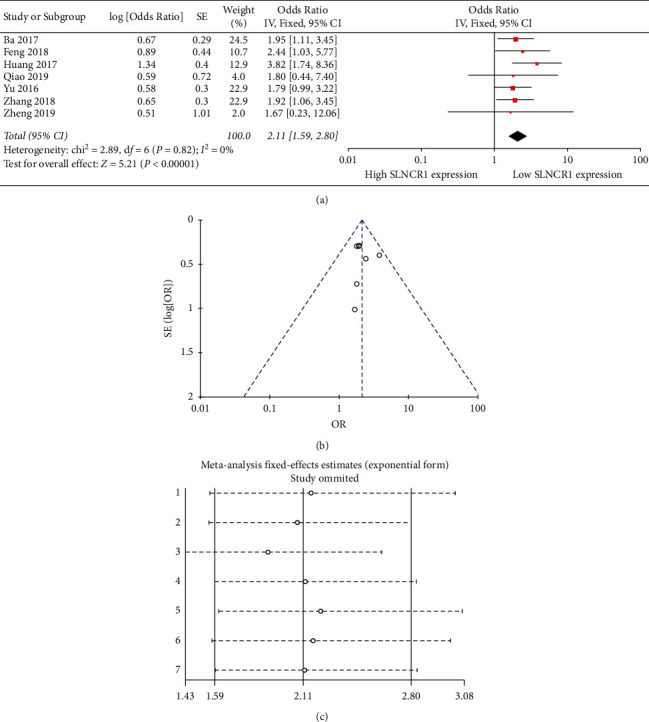
The relation between SLNCR1 expression and overall survival. (a) Forest plot. (b) Funnel plot. (c) Sensitivity analysis.

A meta-analysis was performed to estimate the relationship between SLNCR1 expression and OS. HR was extracted from the survival curves in 7 [14, 17–19, 21–23] of the studies. As shown in Figure 2(a), a fixed-effect model was used since no significant heterogeneity was observed (*I*^2^ = 0, *P*=0.82). The combined HR was 2.11 (95%nCI: 1.59–2.80, *P* < 0.00001), revealing that OS in cancers was markedly related to SLNCR1 expression, with the high SLNCR1 expression group displaying poorer OS than the low SLNCR1 expression group. No obvious asymmetry was detected by the shape of the funnel plot (Figure 2(b)). Sensitivity analysis demonstrated no significant influence by eliminating any single study on the pooled HR, revealing that the results were stable (Figure 2(c)).

**Figure 3:**
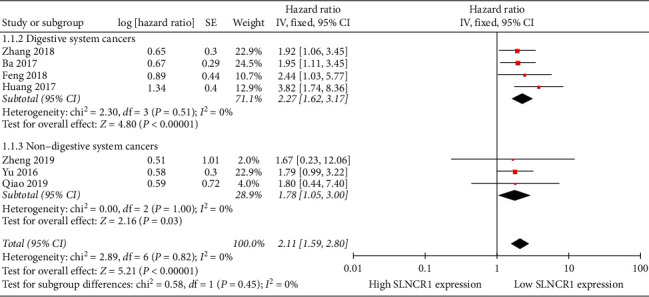
Forest plot for the relation between SLNCR1 expression and overall survival based on different types of cancers.

Two subgroups (digestive system cancers and nondigestive system cancers) were established to assess the HR among different types of cancer (Figure 3). The result suggests that high SLNCR1 expression was associated with poor OS (digestive system cancers: HR: 2.27, 95% CI: 1.62–3.17, *I*^2^ = 0%, *P* < 0.00001; nondigestive system cancers: HR: 1.78, 95% CI: 1.05–3.00, *I*^2^ = 0%, *P*=0.03) regardless of the subgroup (see combined HR data above).

### 3.3. Association between SLNCR1 Expression and Clinicopathological Characteristics

**Figure 4:**
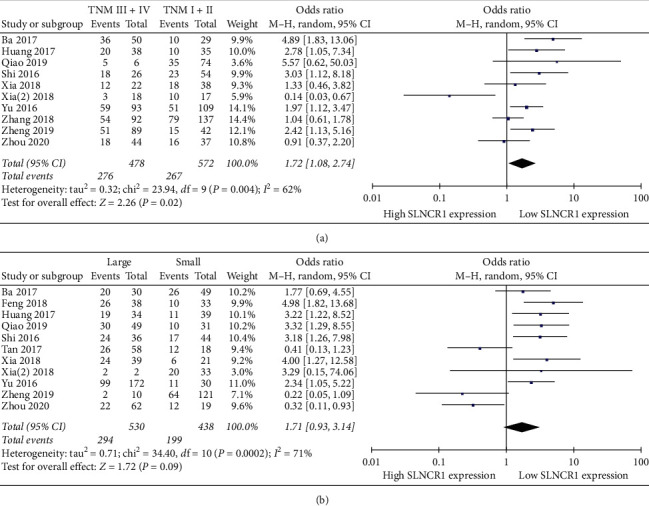
Forest plot for the relation between SLNCR1 expression and clinicopathological characteristics. (a) TNM. (b) Tumor size.

10 [15, 17–25] and 11 [14–21, 23–25] eligible studies reported the state of TNM and tumor size, respectively, based on the expression level of SLNCR1. Compared with the low expression group, the high expression group displayed more advanced TNM stages (OR: 1.72, 95% CI: 1.08–2.74, *I*^2^ = 62%, *P*=0.02) (Figure 4(a)) with respect to SLNCR1 expression. However, no relationship was found between elevated expression of SLNCR1 and tumor sizes (OR: 1.71, 95% CI: 0.93–3.14, *I*^2^ = 71%, *P*=0.09) (Figure 4(b)). Other relationships between SLNCR1 expression and clinicopathological characteristics were uninvestigated because of insufficient data.

### 3.4. Association between SLNCR1 Expression and Metastasis

**Figure 5:**
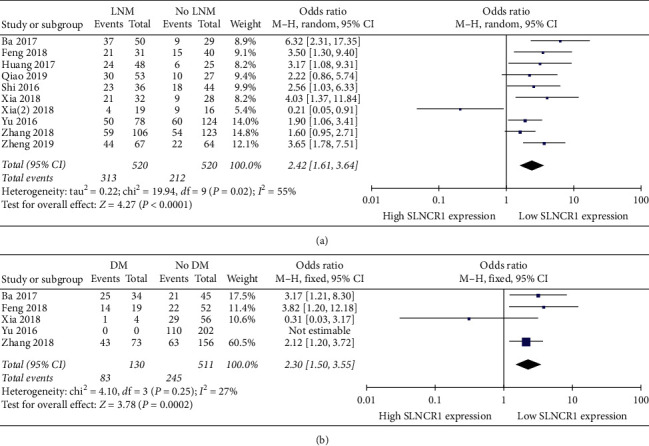
Forest plot for the relation between SLNCR1 expression and metastasis. (a) LNM. (b) DM.

Data regarding the association between SLNCR1 expression and LNM were collected from the 12 [14–25] eligible studies. The random-effect model was adopted because of significant heterogeneity, and high SLNCR1 expression was correlated with LNM (OR: 2.42, 95% CI: 1.61–3.64, *I*^2^ = 55%, *P* < 0.0001) (Figure 5(a)). Furthermore, a relationship between SLNCR1 expression and DM was detected in five [14, 18, 20–22] studies for which the fixed-effect model was used based on limited heterogeneity. Subsequently, a significant difference was found between high SLNCR1 expression and DM (OR: 2.30, 95% CI: 1.50–3.55, *I*^2^ = 27%, *P*=0.0005) (Figure 5(b)).

### 3.5. Association between SLNCR1 Expression and Clinical Features

**Figure 6:**
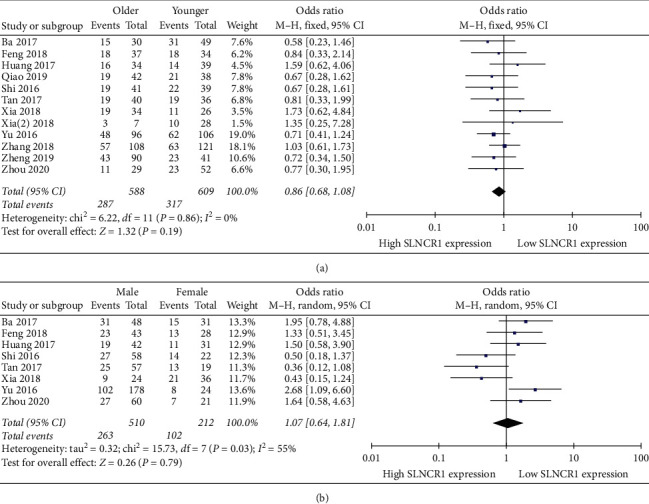
Forest plot for the relation between SLNCR1 expression and clinical features. (a) Age. (b) Gender.

The association between SLNCR1 expression and age was examined in 12 [14–25] eligible studies. A fixed-effect model was utilized to calculate the OR because no significant heterogeneity was observed among the enrolled studies.  Figure 6(a) shows that elevated expression of SLNCR1 was not correlated with age (OR: 0.86, 95% CI: 0.68–1.08, *I*^2^ = 0%, *P*=0.19). Moreover, only 8 [14–16, 18–21, 25] studies described in detail the relationship between SLNCR1 expression and gender. As shown in Figure 6(b), no correlation was found between elevated expression of SLNCR1 and gender (OR: 1.07, 95% CI: 0.64–1.81, *I*^2^ = 55%, *P*=0.79).

### 3.6. Publication Bias and Sensitivity Analysis

**Figure 7:**
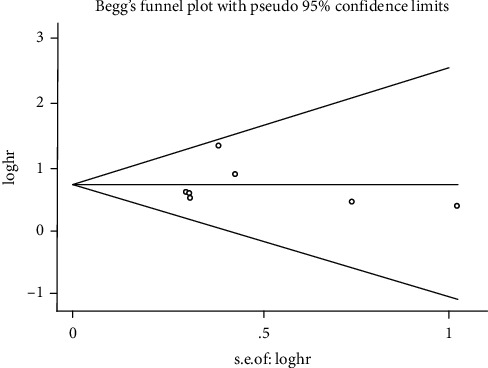
Begg's funnel plot for the evaluation of potential publication bias in the impact of SLNCR1 on overall survival.

The publication bias of this meta-analysis was estimated by Begg's and Egger's tests (Figure 7). No evidence of publication bias was found in the meta-analysis of OS by Begg's (*P*=0.54) or Egger's (*P*=0.80) test. However, sensitivity analysis by elimination of each study to determine its effect on the calculation of overall risk of disease found that two studies significantly affected the analysis of the relationship between SLNCR1 expression and TNM stage. After omitting the study by Ba et al. [18], the overall risk became worthy of suspicion, the OR was reduced from 1.85 (95% CI: 1.12–3.06) to 1.65 (95% CI: 0.99–2.73), and strong heterogeneity persisted (from *I*^2^ = 64% to *I*^2^ = 60%). Additionally, the study by Yu et al. [21] deeply impacted the overall risk in the TNM analysis group. The overall risk changed from OR 1.72 (95% CI: 1.08–2.74) to 1.67 (95% CI: 0.96–2.92) after excluding this study, and heterogeneity was almost unchanged (from *I*^2^ = 62% to *I*^2^ = 66%).

## 4. Discussion

LncRNAs have been regarded as accidental “transcriptional noise” with little function due to lack of protein-coding capability [26]. Recently, accumulating evidence has shown that LncRNAs may regulate genes or miRNA expression and act as oncogenic or tumor suppressors [27, 28]. Some LncRNAs have been shown to act as competing endogenous RNA by regulating miRNA target genes indirectly. With the development of high-throughput genome sequencing technologies, LncRNAs have been identified as new biomarkers for the accurate prognosis of various kinds of tumors due to their functions in tumor proliferation, invasion, migration, and metastasis.

SLNCR1, a LncRNA with high accuracy prognostic value, has been demonstrated to be associated with tumorigenesis and progression and was initially associated with decreased melanoma patient survival. Brain-specific homeobox protein 3a and androgen receptors bind within SLNCR1's conserved region, activating matrix metalloproteinase 9 and subsequently increasing malignant melanoma invasion [6]. Furthermore, SLNCR1 may regulate cell migration, invasion, and stemness through interactions with secretory sPLA2 in nonsmall cell lung cancer [29]. It has been shown to promote the proliferation of breast cancer by sponging miR-515-5p to regulate MARK4 expression and inhibit the Hippo signaling pathway [23]. An increasing number of studies have explored SLNCR1 interaction partners and biological functions in various types of cancers, but the relationship between the expression of SLNCR1 and tumor progression remains poorly understood.

The current study presents the first meta-analysis to evaluate the relationship between SLNCR1 expression and the prognosis of cancers. The results indicate that patients with high expression levels of SLNCR1 tend to have poorer OS than those with low expression levels. In other words, the high expression level of SLNCR1 is a predictor of a negative prognosis of cancer. Meanwhile, subgroup analysis in the fixed model was performed to assess the role of SLNCR1 in digestive system cancers. The data show that in both digestive and nondigestive system tumors, high SLNCR1 expression was associated with poor prognosis. Furthermore, the results also indicate that tumor stage and the high SLNCR1 expression group were markedly higher, and high SLNCR1 expression was correlated with greater susceptibility to LNM and DM. No relationship between SLNCR1 expression and tumor size and clinical features (age and gender) was observed. Moreover, the cutoff values varied among different studies, which might have caused heterogeneity in the results. In order to clarify the source of heterogeneity, we divided the comparison into subgroups with different cutoff values and analyzed the heterogeneity. As shown in Supplementary Materials (available here), the results showed no heterogeneity changes in OS, TNM, tumor size, LNM, DM, age, and gender. Thus, the cause of heterogeneity remains unclear.

Several limitations regarding this meta-analysis should be taken into account. Initially, the HRs and 95% CIs were extracted from Kaplan–Meier curves; lacking sufficient survival data may have led to extraneous heterogeneity. Second, all of the patients were from China, and the results may not represent the global population.

Furthermore, only 12 studies with 1155 patients were involved in the present meta-analysis. Thus, the small sample size of the study may have reduced the stringency of the conclusion. Finally, age and tumor size were defined by the ranges given in the included studies, and different studies had varying criteria for evaluating these parameters. Thus, more rigorous research studies are needed to confirm our conclusions.

In summary, high SLNCR1 expression was associated with poor OS, advanced tumor stage, LNM, and DM in multiple cancers. Thus, the results of our meta-analysis indicate that SLNCR1 may serve as a prognosis biomarker for cancer patients in China.

## Figures and Tables

**Table 1:** Characteristics of studies included in the meta-analysis.

Study (year)	Region	Cancer type	Sample size	Age (low/high)	Male (low/high) Female (low/high)	Tumor size (low/high)	LNC00673 high expression			LNC00673 low expression			TNM (low/high)	HR (95% CI)	Cutoff	Method	NOS
							Total	LNM	DM	Total	LNM	DM					
Feng (2018) [14]	China	CRC	71	>60 (19/18) ≤60 (16/18)	20/23 15/13	≤5 cm (23/10) >5 cm (12/26)	36	21	14	35	10	5	—	2.43 (1.02–5.81)	Median	Q-PCR	6
Shi (2016) [15]	China	NSCLC	80	<65 (17/22) ≥65 (22/19)	31/27 8/14	<5 cm (27/17) ≥5 cm (12/24)	41	23	—	39	13	—	I + II (31/23) III + IV (8/18)	—	NR	Q-PCR	6
Tan (2017) [16]	China	NSCLC	76	>60 (17/19) ≤60 (21/19)	32/25 6/13	≤3 cm (6/12) >3 cm (32/26)	—	—	—	—	—	—	—	—	Median	Q-PCR	6
Zheng (2019) [17]	China	EOC	131	<50 (18/23) ≥50 (47/43)	—	<1 cm (57/64) ≥1 cm (8/2)	66	44	—	65	23	—	I + II (27/15) III + IV (38/51)	1.66 (0.23–11.91)	Median	Q-PCR	7
Ba (2017) [18]	China	GC	79	≤55 (18/31) >55 (15/15)	17/31 16/15	<5 cm (23/26) >5 cm (10/20)	46	37	25	33	13	9	I + II (19/10) III + IV (14/36)	1.95 (1.11–3.45)	Median	Q-PCR	6
Huang (2017) [19]	China	GC	73	>65 (18/16) ≤65 (25/14)	23/19 20/11	≤5 cm (28/11) >5 cm (15/19)	30	24	—	40	24	—	I + II (25/10) III + IV (18/20)	3.81 (1.74–8.32)	FC > 2	Q-PCR	6
Xia (2018) [20]	China	THCA	60	≤45 (15/11) >45 (15/19)	15/9 15/21	≤10 cm (15/6) >10 cm (15/24)	30	21	1	30	11	3	I + II (20/18) III + IV (10/12)	—	Median	Q-PCR	7
Yu (2016) [21]	China	TSCC	202	≤50 (44/62) >50 (48/48)	76/102 16/8	T1 (19/11) T2–T4 (73/99)	110	50	0	92	28	0	I + II (58/51) III + IV (34/59)	1.79 (1.00–3.21)	NR	Q-PCR	6
Zhang (2018) [22]	China	PC	229	>60 (51/57) ≤60 (58/63)	—	—	120	59	43	109	47	30	I + II (58/79) III + IV (38/54)	1.91 (1.06–3.46)	NR	Q-PCR	7
Qiao (2019) [23]	China	BC	80	<50 (17/21) ≥50 (23/19)	—	≤2 cm (21/10) >2 cm (19/30)	40	30	—	40	23	—	I + II (39/35) III + IV (1/5)	1.81 (0.44–7.46)	NR	Q-PCR	6
Xia (2018) [24]	China	BC	35	≤60 (10/18) >60 (3/4)	—	≤5 cm (13/20) >5 cm (0/2)	22	15	—	13	4	—	I + II (10/7) III + IV (3/15)	—	Median	Q-PCR	6
Zhou (2020) [25]	China	ESCC	39	>60 (18/11) ≤60 (29/23)	33/27 14/7	≤5 cm (7/12) >5 cm (40/22)	—	—	—	—	—	—	I + II (21/16) III + IV (26/18)	—	Median	Q-PCR	7

CRC, colorectal cancer; NSCLC, nonsmall cell lung cancer; EOC, epithelial ovarian cancer; GC, gastric cancer; THCA, thyroid carcinoma; TSCC, tongue squamous cell carcinoma; PC, pancreatic cancer; BC, breast cancer; LNM, lymph node metastasis; DM, distant metastasis; HR, hazard ratio; NR, not reported; FC, fold change; qRT-PCR, quantitative real-time-polymerase chain reaction; NOS, Newcastle–Ottawa Scale.

## Data Availability

The data used to support the findings of this study are available from the corresponding author upon request.

## Abbreviations

SLNCR1: Steroid receptor RNA activator (SRA)-like noncoding RNA
HR: Hazard ratio
lncRNA: Long noncoding RNA
OR: Odds ratio
OS: Overall survival
LNM: Lymph node metastasis
DM: Distant metastasis.

## Supplementary Materials

Supplementary Materials Subgroups were established to analyze the heterogeneity according to different cutoff values.
