# Supplementary figures and images for: The Prognostic Value of LncRNA SLNCR1 in Cancers: A Meta-Analysis
Source: J Oncol. 2021 Oct 25;2021:3161714. doi: 10.1155/2021/3161714 (PMC8560271; doi:10.1155/2021/3161714)

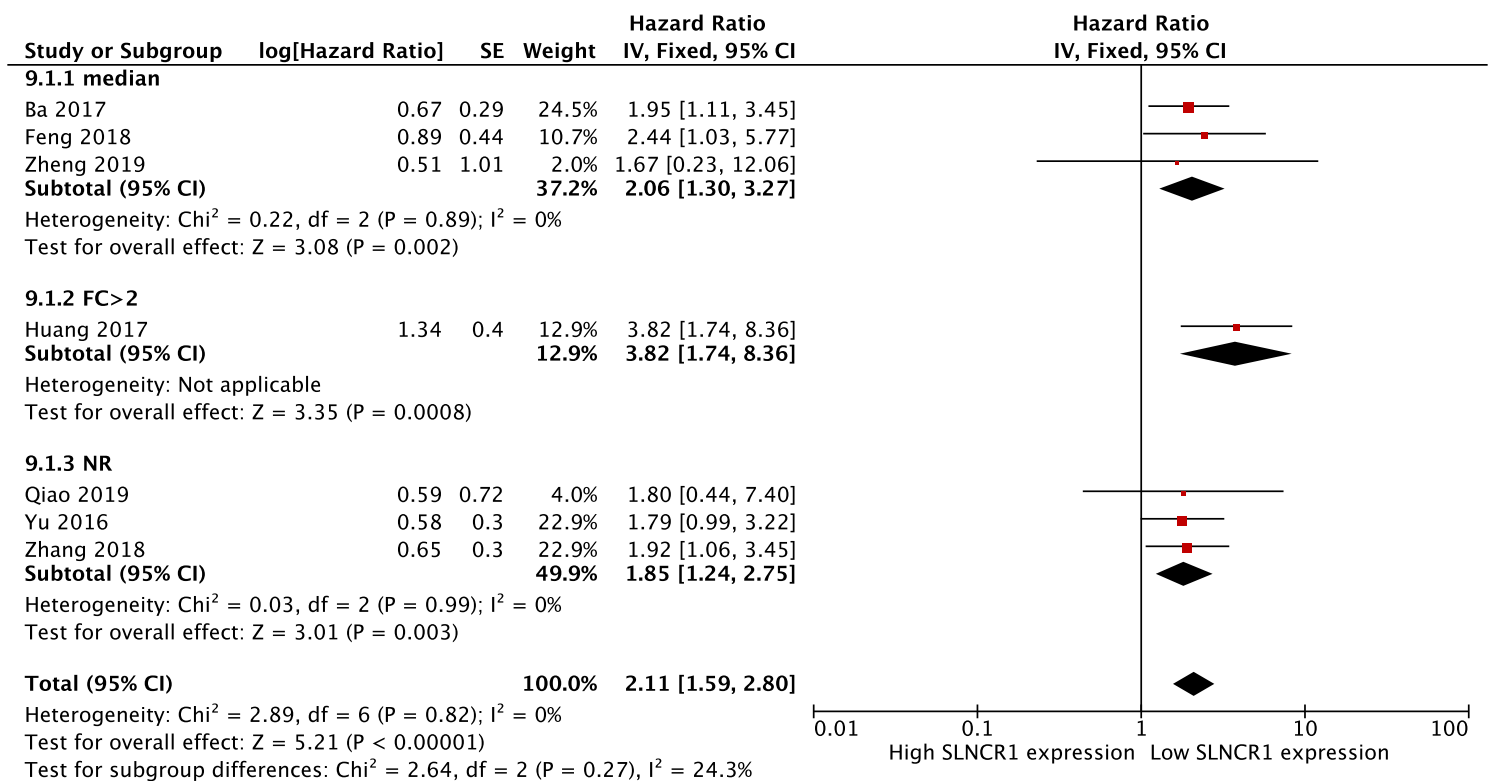

Supplement: Supplementary Materials — Subgroups were established to analyze the heterogeneity according to different cutoff values. [file 3161714.f1.zip › 3161714.f1/Supplementary figure 1.pdf]

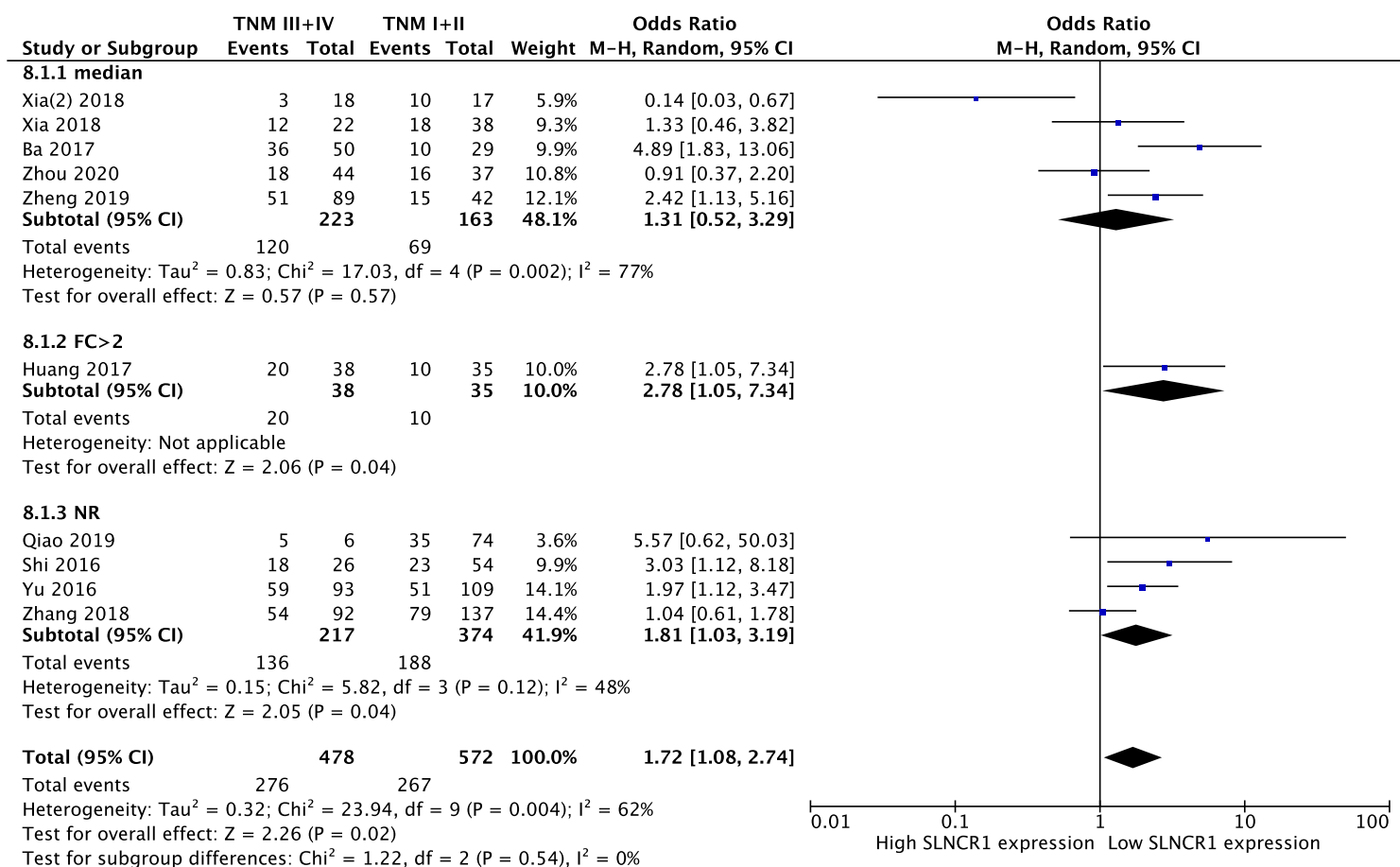

Supplement: Supplementary Materials — Subgroups were established to analyze the heterogeneity according to different cutoff values. [file 3161714.f1.zip › 3161714.f1/Supplementary figure 2.pdf]

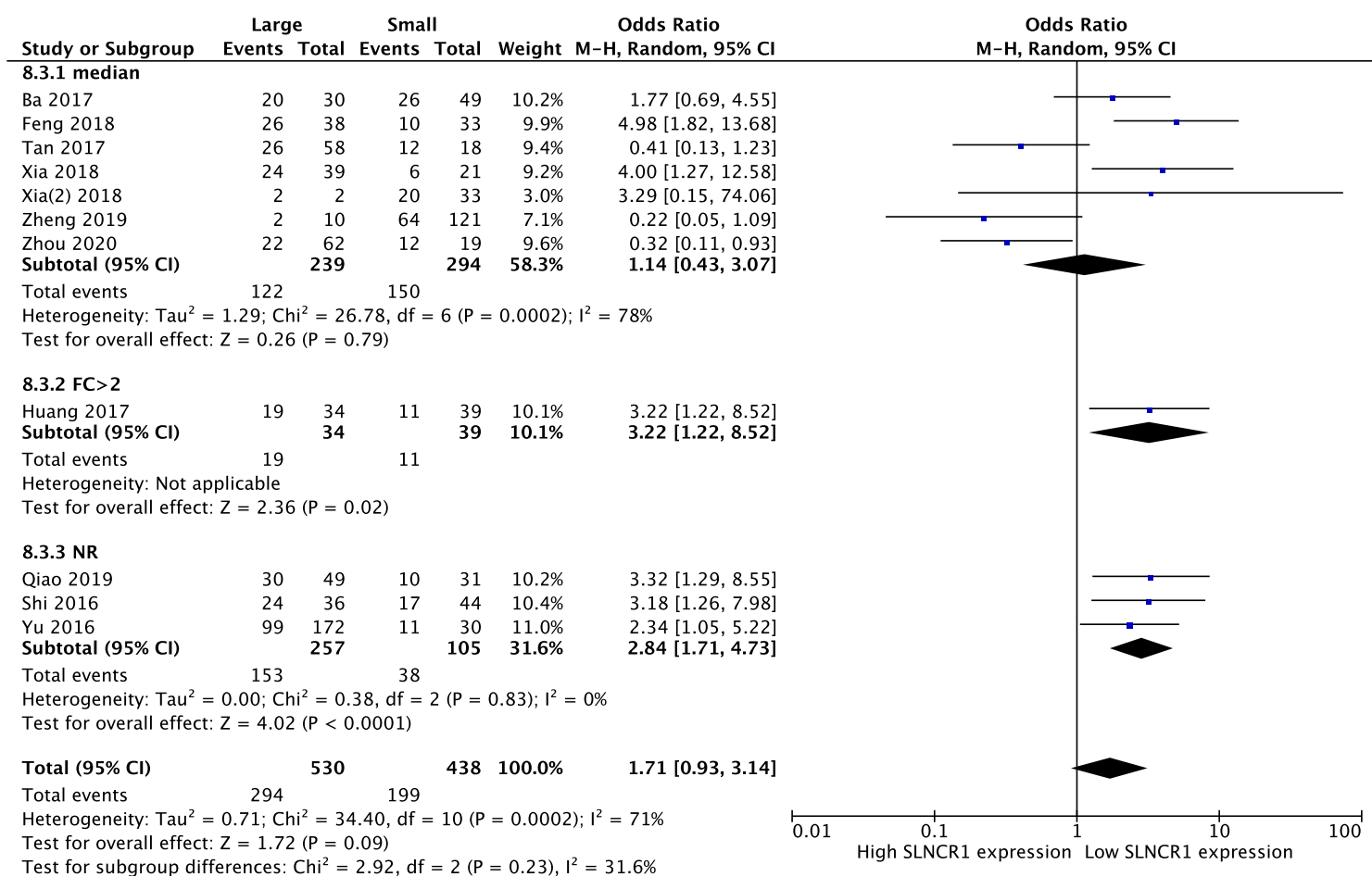

Supplement: Supplementary Materials — Subgroups were established to analyze the heterogeneity according to different cutoff values. [file 3161714.f1.zip › 3161714.f1/Supplementary figure 3.pdf]

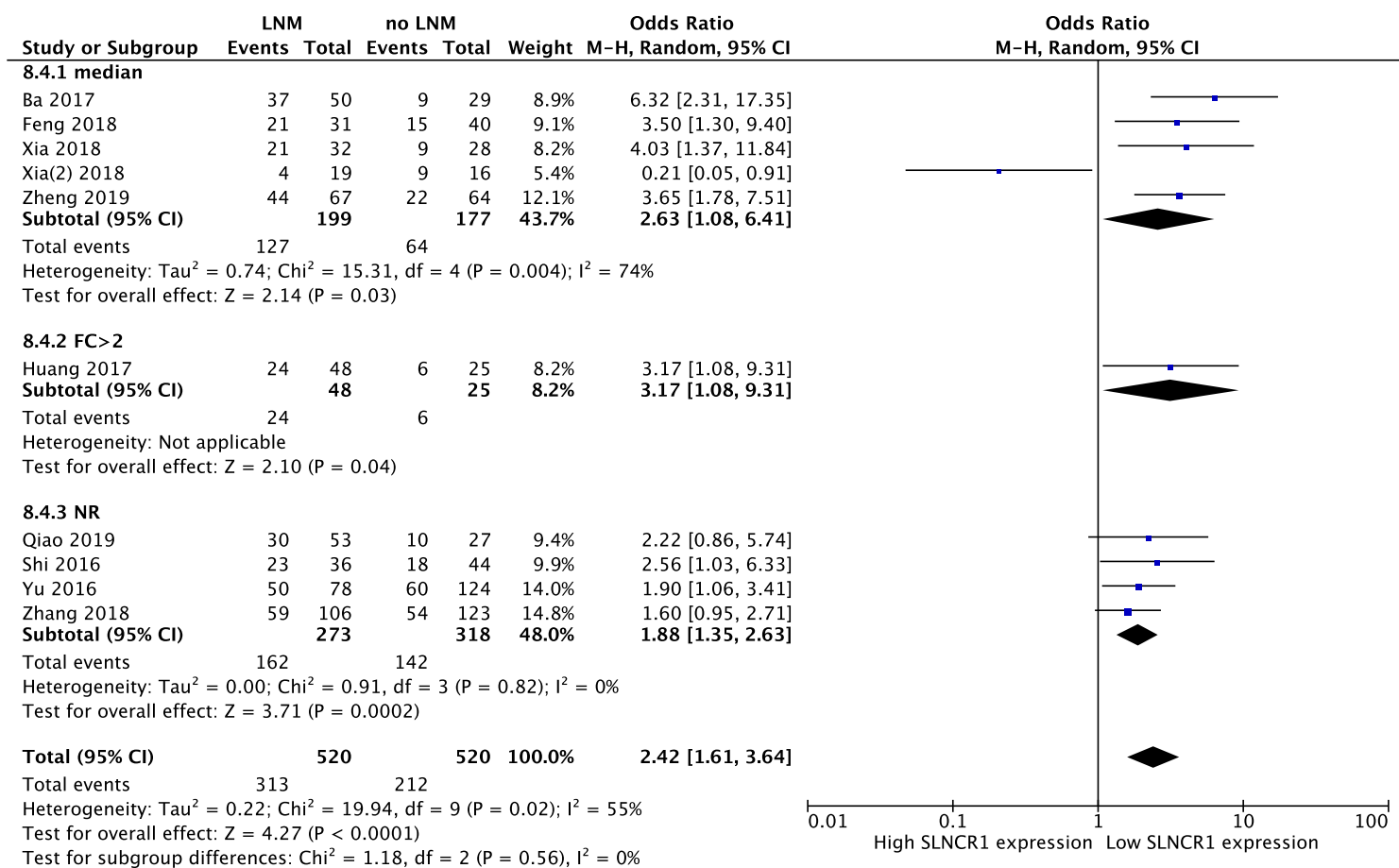

Supplement: Supplementary Materials — Subgroups were established to analyze the heterogeneity according to different cutoff values. [file 3161714.f1.zip › 3161714.f1/Supplementary figure 4.pdf]

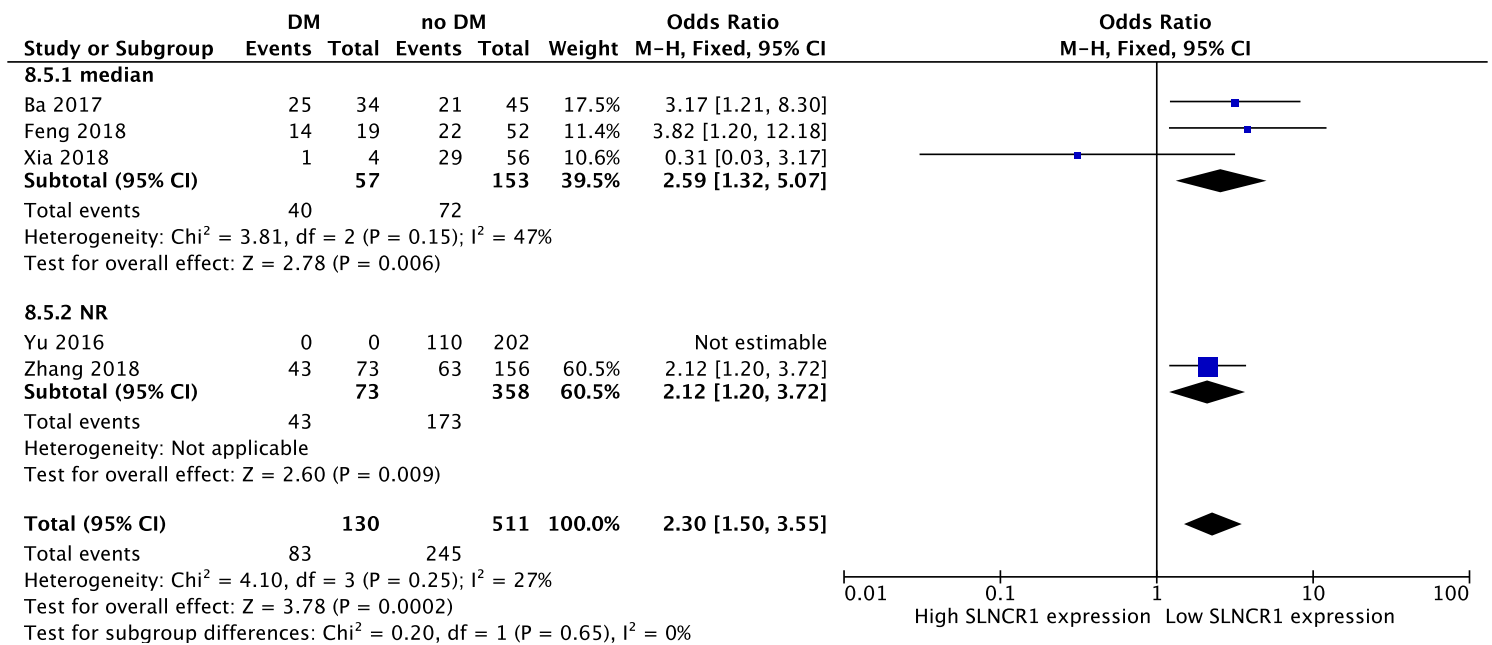

Supplement: Supplementary Materials — Subgroups were established to analyze the heterogeneity according to different cutoff values. [file 3161714.f1.zip › 3161714.f1/Supplementary figure 5.pdf]

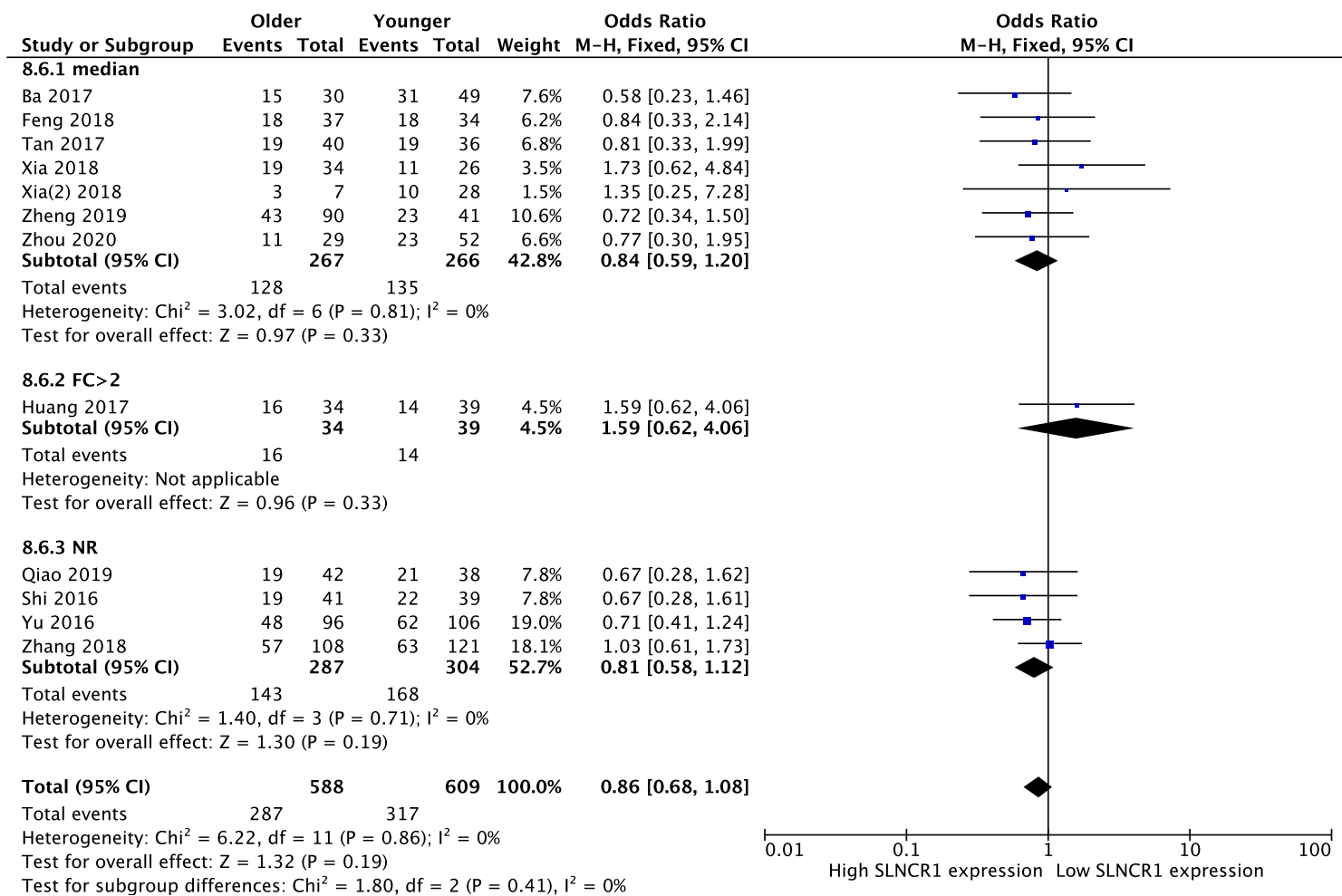

Supplement: Supplementary Materials — Subgroups were established to analyze the heterogeneity according to different cutoff values. [file 3161714.f1.zip › 3161714.f1/Supplementary figure 6.pdf]

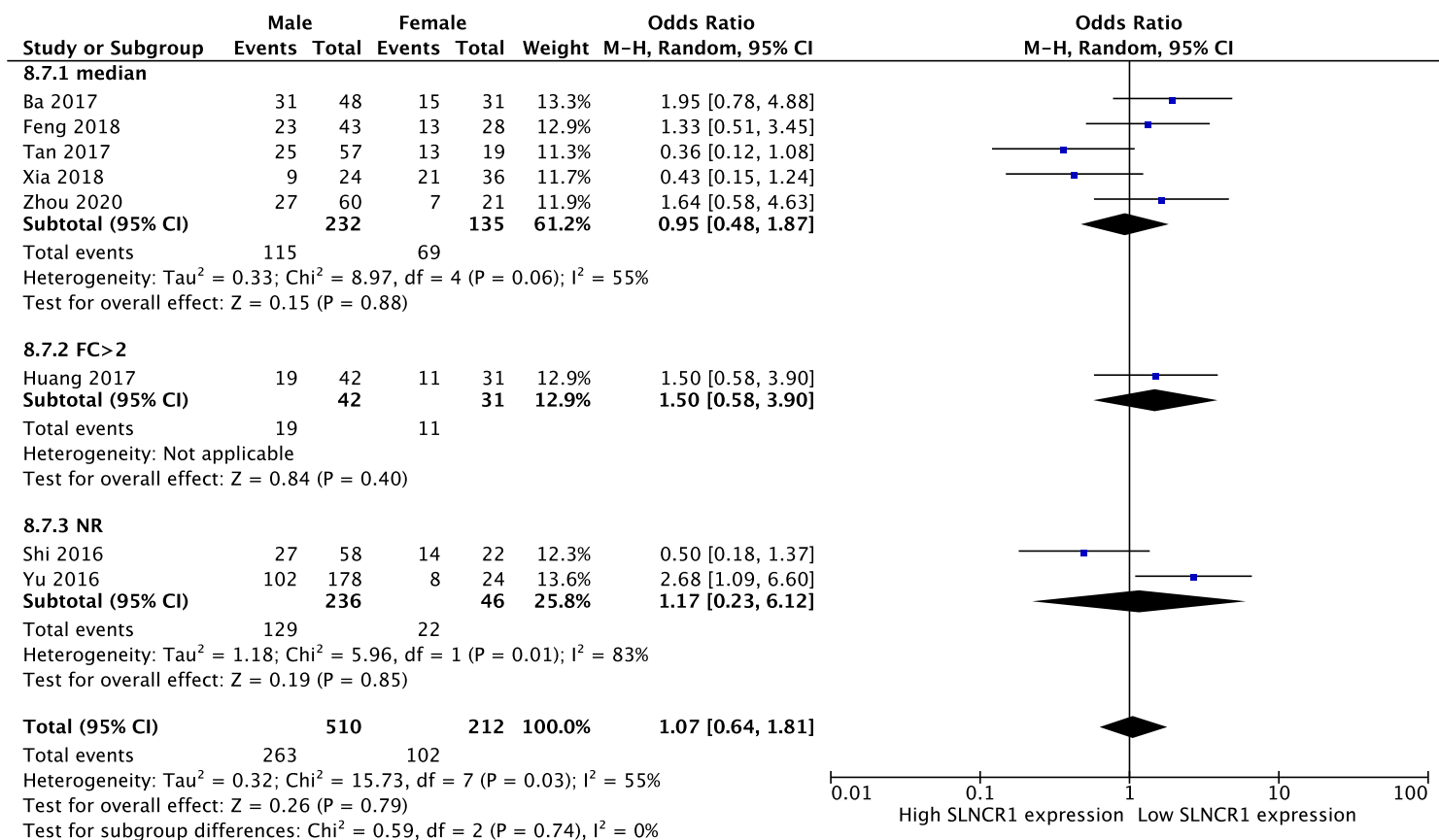

Supplement: Supplementary Materials — Subgroups were established to analyze the heterogeneity according to different cutoff values. [file 3161714.f1.zip › 3161714.f1/Supplementary figure 7.pdf]
